# Effect of dual-rotation on MHD natural convection of NEPCM in a hexagonal-shaped cavity based on time-fractional ISPH method

**DOI:** 10.1038/s41598-021-02046-z

**Published:** 2021-11-22

**Authors:** Zehba Raizah, Abdelraheem M. Aly

**Affiliations:** 1 Department of Mathematics, Faculty of Science, King Khalid University, Abha, 62529 Saudi Arabia; 2 Department of Mathematics, Faculty of Science, South Valley University, Qena, 83523 Egypt

**Keywords:** Mechanical engineering, Applied mathematics

## Abstract

The time-fractional derivative based on the Grunwald–Letnikove derivative of the 2D-ISPH method is applying to emulate the dual rotation on MHD natural convection in a hexagonal-shaped cavity suspended by nano-encapsulated phase change material (NEPCM). The dual rotation is performed between the inner fin and outer hexagonal-shaped cavity. The impacts of a fractional time derivative $$\alpha$$ $$\left( {0.92 \le \alpha \le 1} \right)$$, Hartmann number Ha $$\left( {0 \le Ha \le 80} \right)$$, fin length $$\left( {0.2 \le L_{Fin} \le 1} \right)$$, Darcy parameter Da $$\left( {10^{ - 2} \le Da \le 10^{ - 4} } \right)$$, Rayleigh number Ra $$\left( {10^{3} \le Ra \le 10^{6} } \right)$$, fusion temperature $$\theta_{f}$$ $$\left( {0.05 \le \theta_{f} \le 0.8} \right)$$, and solid volume fraction $$\varphi$$ $$\left( {0 \le \varphi \le 0.06} \right)$$ on the velocity field, isotherms, and mean Nusselt number $$\overline{Nu}$$ are discussed. The outcomes signaled that a dual rotation of the inner fin and outer domain is affected by a time-fractional derivative. The inserted cool fin is functioning efficiently in the cooling process and adjusting the phase change zone within a hexagonal-shaped cavity. An increment in fin length augments the cooling process and changes the location of a phase change zone. A fusion temperature $$\theta_{f}$$ adjusts the strength and position of a phase change zone. The highest values of $$\overline{Nu}$$ are obtained when $$\alpha = 1$$. An expansion in Hartmann number $$Ha $$ reduces the values of $$\overline{Nu}$$. Adding more concentration of nanoparticles is improving the values of $$\overline{Nu}$$.

## Introduction

In the heat and mass transfer field, scientists and researchers aimed to find solutions analytically, numerically, or experimentally for analyzing the thermal environments and explaining physical phenomena. Also, simulating the heat convection and conduction processes. Experimental solutions usually take longer and cost more than analytical solutions, and therefore they resorted to many different analytical and numerical methods to model these phenomena at a lower cost in a short time and allows studying many variables and parameters of the issue under study. The convection inside a complex-shaped cavity within heated fins supplied with a nano-encapsulated phase change material (NEPCM) has taken the researchers attention as an effective way to enhancement the heat and mass transfer and solving mechanical engineering problems.

### The nano-encapsulated phase change material (NEPCM)

The NEPCMs consist of the core and the shell. The cores are made of a Phase Change Material (PCM). It solidifies or melts at a special temperature called s fusion temperature. The shell consists of a polymer. The NEPCM suspensions are a new form of hybrid nanofluids. It has several heat transfer applications^1,2^. The convection of heat and fluid flow of NEPCM have been reported in wide theoretical studies such as enclosures^3–6^, divergent heatsink^7,8^, energy storage^9^, with fins^10^, and others^11,12^. Shafee et al.^13^ used Galerkin approach of the finite element method to handle the phase change process of NEPCM in a heat storage. Selimefendigil et al.^14^ analyzed numerically the natural convection of CuO–water nanofluid in a square cavity with a conductive partition and a phase change material (PCM) under the effect of a uniform inclined magnetic field. The mixed convection in a phase change material-filled a square cavity under the effect of a rotating cylinder was numerically investigated by Selimefendigil and Öztop^15^. In further studies, Selimefendigil et al.^16,17^ presented different numerical studies on the phase change dynamics of a 3D cylinder containing hybrid nanofluid and phase change material (PCM) by using the finite element solver.

### Internal fins

As mentioned in the previous part, the use of phase change material (PCM) has acquired highly increasing concern in various engineering applications, through thermal administration systems, solar energy storage, and conservation of the energy in buildings^18,19^. But the PCMs have low thermal conductivity obstruct the heat transfer during solidification or melting processes, which break down the efficiency of energy storage. So, different methods have been suggested to enhance the heat transfer of the PCM. One of them is adding the internal fins. Different geometric parametric of the internal fins like their thickness, length, location, and inclination angle have been investigated. Ren and Chan^20^ found that the longer fins were more active than the shorter fins to increase the melting rate of PCM. Sciacovelli et al.^21^ examined differently shaped fins to enhance the heat inside the cylindrical cavity. They reported that the tree-shaped fin was increased the system energy efficiency by 24%. Ji et al.^22^ studied the effects of fins inclination angles on the melting rate of PCM. Li and Yu^23^ investigated the influence of the internal fins on the melting process, the design of dual fins, and tree-shaped fins. They illustrated how changing the fins structures better than increasing the number of fins in the offers a higher rate of heat transfer and a better energy storage capacity. More different configurations of fixed or flexible fins, and their design and geometric factors can be found in these studies^24–29^.

### Rotating cavity

The study of heat and fluid flow inside rotating cavities is significant from both theoretical as well as application sides of view. These kinds of studies are more complex due to the rotation, and different body forces have driven the flow. As examples of its applications, in astrophysical and geophysical flows, for semiconductors in manufacturing of single wafer crystal, to storage the thermal energy in rotating systems, to cooling the tools microelectronic, and so on. Earlier studies were presented to investigate the effects of the fluid flow on the flow and heat transfer rate inside the rotating enclosures^30–38^. Mandal and Sonawane^39^ studied the flows inside a differentially heated rotating square cavity in two different formulations. They found that the force of inertia appears effect by the increasing speed of the enclosure rotation. Other studies of natural convection and surface radiation in a rotating square cavity with low rotation velocity were presented by Mikhailenko et al.^40–43^.

### The fractional derivatives

When the order of the derivatives and integrals are non-integers in the classical partial differential equations, then we have a new kind of differential equations called fractional differential equations (FPDEs). The importance of fractional differential equations comes from their wide applications in engineering and science. These equations can be used to simulate the problems in fluid dynamics, electrochemistry, electrodynamics, nanotechnology, astronomy sciences, and chemical physics.

We can summarize the famous types of fractional derivatives as:

1. Grunwald–Letnikove derivative:

This type was firstly presented in 1867 by Anton Karl Grünwald, then by Aleksey Vasilievich Letnikov. It takes the form^44^:

$$ \begin{aligned} & D_{t}^{\alpha } f\left( t \right) = \mathop {\lim }\limits_{\Delta t \to 0} \frac{1}{{\left( {\Delta t} \right)^{\alpha } }} \mathop \sum \limits_{m = 0}^{n} \left( { - 1} \right)^{m} \left( {\alpha m } \right) f\left( {t - m \Delta t} \right), \\ & t \ge 0, \left( {\alpha m } \right) = \frac{{\Gamma \left( {\alpha + 1} \right)}}{{\Gamma \left( {m + 1} \right)\Gamma \left( {\alpha - m + 1} \right)}} \\ \end{aligned} $$

2. Riemann–Liouville fractional derivative:

Which was presented by Riemann in 1847, and take the form:

$$ D_{t}^{\alpha } f\left( t \right) = \frac{1}{{\Gamma \left( {n - \alpha } \right)}}\frac{{d^{n} }}{{dt^{n} }}\mathop \int \limits_{a}^{t} \frac{f\left( x \right)}{{\left( {t - x} \right)^{\alpha - n + 1} }}dx, \quad 0 < \alpha < n $$

3. Caputo derivative:
  Caputo derivative^45^ was acquired in 1967. It was defined as:
  $$ D_{t}^{\alpha } f\left( t \right) = \frac{1}{{\Gamma \left( {n - \alpha } \right)}}\mathop \int \limits_{a}^{t} \frac{{D^{n} f\left( \eta \right)}}{{\left( {t - \eta } \right)^{\alpha - n + 1} }}d\eta , \quad n - 1 < \alpha < 1 $$
4. The conformable fractional derivative

Which presented by Khalil et al.^46^ in 2014:

$$ D_{t}^{\alpha } f\left( t \right) = \mathop {\lim }\limits_{{\varepsilon^{*} \to 0}} \frac{{f\left( {t + \varepsilon^{*} t^{1 - \alpha } } \right) - f\left( t \right)}}{{\varepsilon^{*} }}, \quad 0\left\langle {\alpha \le 1, t} \right\rangle 0 $$

From these types, the researchers choose the type, which is more compatible with the experimental results, when they want to solve the system's equations of the physical problems.

Many good studies were presented and published to illustrate the applications of fractional calculus to transport processes^47–52^. In general, the mesh-free nature of the ISPH method helps in handling the high deformation and fluid–structure interaction problems. So, the ISPH method is adopted for the current problem of the rotating paddle wheel inside a novel geometry of a cross-shaped cavity. The objective of this investigation is to employ the time-fractional derivative in the solving steps of the ISPH method. The dual rotation and inclined magnetic impacts on the natural convection of NEPCM embedded in a hexagonal-shaped cavity are conducted. The dual rotation between an inner fin and outer hexagonal-shaped cavity during natural convection flow can be applied in generating thermal energy from the rotating systems and cooling process of the electronic devices. The results indicated that the fractional time derivative changes the dual rotation between the inner fin and outer domain. The inserted cool fin is functioning efficiently in the cooling process and adjusting the phase change zone within a hexagonal-shaped cavity. A fusion temperature alters the strength and position of a phase change zone. The highest values of $\overline{Nu}$ are found at higher value of the time-fractional derivative $\left( {\alpha = 1} \right)$. Increasing Hartmann number reduces the values of $\overline{Nu}$, whilst increasing solid volume fraction enhances the values of $\overline{Nu}$.

## Mathematical analysis

**Figure 1:**
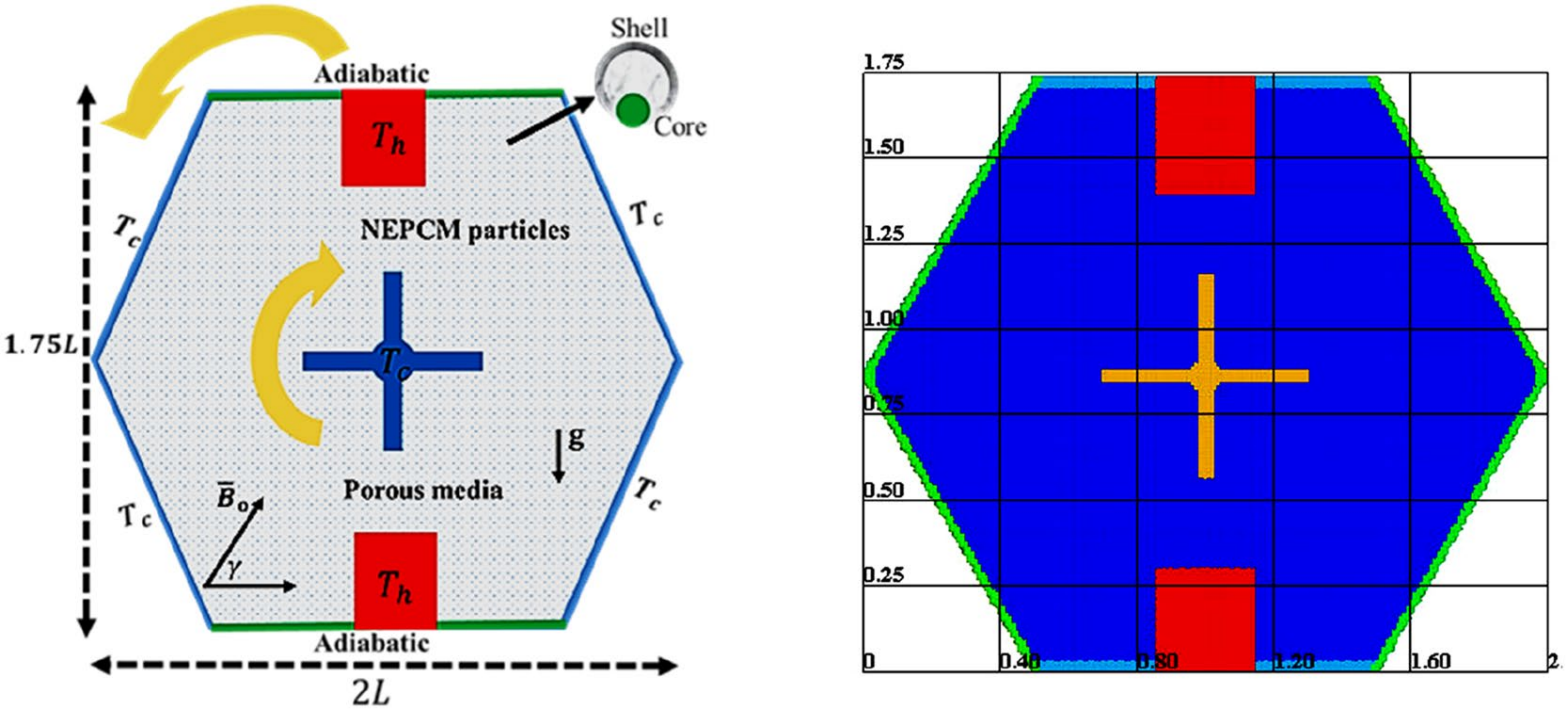
Basic illustration of the present physical problem.

The basic illustration of the present physical problem has been shown in Fig. 1. The inner fin is cooled by a temperature $T_{c}$ and the two rectangles in the flat walls of a cavity is heated by a temperature $T_{h}$. The embedded fin is rotating clockwise, and the outer hexagonal-shaped domain is rotating anticlockwise. It is presumed the latent heat is almost 211 ${\text{kJ}}\left( {{\text{kg}}} \right)^{ - 1}$ whilst the fusion temperature of the PCM cores is about 32 °C. The dual rotation is carrying a uniform circular velocity around the center of a cavity. Table 1 presents the physical attributes of a porous matrix and a mixture fluid. The local thermal equilibrium model is assumed amongst the mixture nanofluid and a porous medium.

**Table 1:** The physical attributes of a mixture fluid^53^.

	Material	$\rho \; \left( {{\text{Kg}}\;{\text{m}}^{ - 3} } \right)$	$\beta \times 10^{ - 5} \;({\text{K}}^{ - 1} )$	$C_{p} \;\left( {{\text{J}}\;{\text{kg}}^{ - 1} \;{\text{K}}^{ - 1} } \right)$	$k \;\left( {{\text{W}}\;{\text{m}}^{ - 1} \;{\text{K}}^{ - 1} } \right)$
Core	Nonadecane	786	17.28	1317.7	
Shell	Polyurethane	721		2037	
Base fluid	Water (25 °C)	997.1	21	4179	0.613
Porous matrix	Glass balls	2700		840	1.05

The dimensional governing equations^53,54^ are:

1 $$ \frac{\partial u}{{\partial x}} + \frac{\partial v}{{\partial y}} = 0 $$

2 $$ \frac{{\rho_{b} }}{\varepsilon }D_{\tau }^{\alpha } u = - \frac{\partial p}{{\partial x}} + \frac{{\mu_{b} }}{\varepsilon }\left( {\frac{{\partial^{2} u}}{{\partial x^{2} }} + \frac{{\partial^{2} u}}{{\partial y^{2} }}} \right) - B_{o}^{2} \delta_{b} \left( {u \sin^{2} \gamma - v \sin \gamma \cos \gamma } \right) - \frac{{\mu_{b} }}{K}u - \frac{1.75}{{\sqrt {150} }}\frac{{\rho_{b} }}{{\sqrt {K\varepsilon^{3} } }}\sqrt {u^{2} + v^{2} } u $$

3 $$ \frac{{\rho_{b} }}{\varepsilon }D_{\tau }^{\alpha } v = - \frac{\partial p}{{\partial y}} + \frac{{\mu_{b} }}{\varepsilon }\left( {\frac{{\partial^{2} v}}{{\partial x^{2} }} + \frac{{\partial^{2} v}}{{\partial y^{2} }}} \right) - B_{o}^{2} \delta_{b} \left( {v \cos^{2} \gamma - u \sin \gamma \cos \gamma } \right) - \frac{{\mu_{b} }}{K}v - \frac{1.75}{{\sqrt {150} }}\frac{{\rho_{b} }}{{\sqrt {K\varepsilon^{3} } }}\sqrt {u^{2} + v^{2} } v + \left( {\rho \beta } \right)_{b} \left( {T - T_{c} } \right)g $$

4 $$ \left[ {\varepsilon \left( {\rho C} \right)_{b} + \left( {1 - \varepsilon } \right)\left( {\rho C} \right)_{s} } \right]D_{\tau }^{\alpha } T = \left( {\varepsilon k_{b} + \left( {1 - \varepsilon } \right)k_{s} } \right)\left( {\frac{{\partial^{2} T}}{{\partial x^{2} }} + \frac{{\partial^{2} T}}{{\partial y^{2} }}} \right) $$

where $\left( {u,v} \right)$ are the dimensional velocity vector, $\varepsilon$ is a porosity, $\rho$ is a density, ${\text{g}}$ is gravity, $\mu$ is a dynamic viscosity, $\beta$ is a thermal expansion, and $K$ is a permeability. Furthermore, Eq. (3) indicates the local thermal equilibrium condition amongst the mixture fluid and the solid matrix.

According to Ghalambaz et al.^53,55,56^, the mixture density is:

5 $$ \rho_{b} = \varphi \rho_{p} + \rho_{f} - \varphi \rho_{f} $$

where $\rho_{p}$ is a density of NEPCM particles. $\rho_{p}$ is calculated from a density of a core $\rho_{c}$ and a shell $\rho_{s}$ as:

6 $$ \rho_{p} = \frac{{\left( {1 + \chi } \right)\rho_{s} \rho_{c} }}{{\rho_{s} + \chi \rho_{c} }} $$

where $\chi \approx 0.447$ is a core–shell weight ratio for NEPCMs.

The Specific heat capacity is calculated as:

7 $$ (C_{p} )_{b} = \frac{{\varphi \rho_{p} (C_{p} )_{p} + \rho_{f} (C_{p} )_{f} - \varphi \rho_{f} (C_{p} )_{f} }}{{\rho_{b} }} $$

The heat capacity is:

8 $$ (C_{p} )_{p} = \frac{{\left( {(C_{p} )_{c,l} + \chi (C_{p} )_{s} } \right)\rho_{s} \rho_{c} }}{{\left( {\rho_{s} + \chi \rho_{c} } \right)\rho_{p} }} $$

where $(C_{p} )_{c,l}$ and $(C_{p} )_{s}$ are a heat capacity of a core and a shell. The sine profile of the latent heat of phase change is:

9 $$ (C_{p} )_{c} = (C_{p} )_{c,l} + \left[ {\frac{\pi }{2}\left( {\frac{{h_{sf} }}{{T_{Mr} }} - (C_{p} )_{c,l} } \right)\sin \left( {\pi \frac{{T - T_{f} + \frac{{T_{Mr} }}{2}}}{{T_{Mr} }}} \right) } \right]\Gamma $$

where

10 $$ \Gamma = \left\{ {\begin{array}{*{20}l} 0 \hfill & {T < T_{f} - \frac{{T_{Mr} }}{2}} \hfill \\ 1 \hfill & {\left( {T_{f} - \frac{{T_{Mr} }}{2}} \right) < T < \left( {T_{f} + \frac{{T_{Mr} }}{2}} \right)} \hfill \\ 0 \hfill & {T > T_{f} + \frac{{T_{Mr} }}{2}} \hfill \\ \end{array} } \right., $$

The thermal conductivity, thermal expansion, and dynamic viscosity are:

11 $$ k_{b} = k_{f} \left( {1 + N_{2} \varphi } \right), \beta_{b} = \beta_{f} - \varphi \beta_{f} + \varphi \beta_{p} , \mu_{b} = \mu_{f} \left( {1 + N_{1} \varphi } \right) $$

$N_{1}$ is a dynamic viscosity number and $N_{2}$ is a thermal conductivity number.

The applied dimensionless quantities^54^ are:

12 $$ X = \frac{x}{L}, Y = \frac{y}{L}, U = \frac{uL}{{\zeta_{f} }}, V = \frac{vL}{{\zeta_{f} }},\theta = \frac{{T - T_{c} }}{{T_{h} - T_{c} }}, P = \frac{{pL^{2} }}{{\rho_{f} \zeta_{f}^{2} }}, \tau = \frac{{tL^{2} }}{{\zeta_{f} }}, $$

The dimensionless regulating equations in Lagrangian type^53,54^ are:

13 $$ \frac{\partial U}{{\partial X}} + \frac{\partial V}{{\partial Y}} = 0, $$

14 $$ \frac{1}{\varepsilon }D_{\tau }^{\alpha } U = - \frac{{\rho_{f} }}{{\rho_{b} }}\frac{\partial P}{{\partial X}} + \frac{{\mu_{b} }}{{\varepsilon \mu_{f} }}\frac{{\rho_{f} }}{{\rho_{b} }}\Pr \left( {\frac{{\partial^{2} U}}{{\partial X^{2} }} + \frac{{\partial^{2} U}}{{\partial Y^{2} }}} \right) - \frac{{\sigma_{b} }}{{\sigma_{f} }}\frac{{\rho_{f} }}{{\rho_{b} }}\Pr Ha^{2} \left( {U \sin^{2} \gamma - V \sin \gamma \cos \gamma } \right) - \frac{{\mu_{b} }}{{\mu_{f} }}\frac{{\rho_{f} }}{{\rho_{b} }}\Pr \frac{U}{Da} - \frac{1.75}{{\sqrt {150} }}\frac{1}{{\sqrt {Da\varepsilon^{3} } }}\sqrt {U^{2} + V^{2} } U, $$

15 $$ \frac{1}{\varepsilon }D_{\tau }^{\alpha } V = - \frac{{\rho_{f} }}{{\rho_{b} }}\frac{\partial P}{{\partial Y}} + \frac{{\mu_{b} }}{{\varepsilon \mu_{f} }}\frac{{\rho_{f} }}{{\rho_{b} }}\Pr \left( {\frac{{\partial^{2} V}}{{\partial X^{2} }} + \frac{{\partial^{2} V}}{{\partial Y^{2} }}} \right) + \frac{{\left( {\rho \beta } \right)_{b} }}{{\left( {\rho \beta } \right)_{f} }}\frac{{\rho_{f} }}{{\rho_{b} }}Ra\Pr \theta - \frac{{\sigma_{b} }}{{\sigma_{f} }}\frac{{\rho_{f} }}{{\rho_{b} }}\Pr Ha^{2} \left( {V \cos^{2} \gamma - U \sin \gamma \cos \gamma } \right) - \frac{{\mu_{b} }}{{\mu_{f} }}\frac{{\rho_{f} }}{{\rho_{b} }}\Pr \frac{V}{Da} - \frac{1.75}{{\sqrt {150} }}\frac{1}{{\sqrt {Da\varepsilon^{3} } }}\sqrt {U^{2} + V^{2} } V $$

16 $$ \left[ {\varepsilon Cr + \left( {1 - \varepsilon } \right)\frac{{\left( {\rho C} \right)_{s} }}{{\left( {\rho C} \right)_{f} }}} \right]D_{\tau }^{\alpha } \theta = \frac{{k_{m,b} }}{{k_{f} }}\left[ {\frac{{\partial^{2} \theta }}{{\partial X^{2} }} + \frac{{\partial^{2} \theta }}{{\partial Y^{2} }}} \right], $$

The dimensionless parameters are Raleigh number $Ra = \frac{{\beta_{f} g \left( {T_{h} - T_{c} } \right)L^{3} }}{{\zeta_{f} \nu_{f} }}$, Prandtl number $Pr = \frac{{\nu_{f} }}{{\zeta_{f} }}$, Hartmann number $Ha = \sqrt {\frac{{\sigma_{{{\text{f}} }} }}{{\mu_{{\text{f}}} }}} B_{0} L$, and Darcy parameter $Da = \frac{K}{{L_{ }^{2} }}$.

A uniform circular velocity of a dual rotation is:

The velocities of an outer domain:

17 $$ {\text{U}}_{{{\text{hex}}}} = - \omega \left( {{\text{Y}} - {\text{Y}}_{{\text{o}}} } \right)\quad \& \quad {\text{V}}_{{{\text{hex}}}} = \omega \left( {{\text{X}} - {\text{X}}_{{\text{o}}} } \right) $$

The velocities of an inner fin:

18 $$ {\text{U}}_{fin} = \omega \left( {{\text{Y}} - {\text{Y}}_{{\text{o}}} } \right) \quad \& \quad {\text{V}}_{fin} = - \omega \left( {X - {\text{X}}_{{\text{o}}} } \right) $$

where a dimensionless angular velocity $\omega$ is kept at 2.5.

The boundary conditions:

19 $$ {\text{An}}\;{\text{embedded}}\;{\text{fin}}{:}\;\theta = 0\;\;U = U_{fin} , V = {\text{V}}_{fin} , $$

20 $$ {\text{Rectangle - shapes}}\;{\text{in}}\;{\text{flat}}\;{\text{walls}}\;{\text{of}}\;{\text{a}}\;{\text{cavity}}{:}\;\;\theta = 1,\; U = U_{hex} , V = V_{hex} , $$

21 $$ {\text{Flat}}\;{\text{walls}}\;{\text{of}}\;{\text{a}}\;{\text{cavity}}{:}\;\;\frac{ \partial \theta }{{\partial {\varvec{n}}}} = 0,\; U = U_{hex} , V = V_{hex} , $$

22 $$ {\text{Other}}\;{\text{cavity}}\;{\text{walls}}{:}\;\; \theta = 1,\;U = U_{hex} , V = V_{hex} , $$

The recent studies^53,54,57^ provides the definitions of thermal conductivity $\frac{{k_{m,b} }}{{k_{f} }}$, density ratio $\frac{{\rho_{b} }}{{\rho_{f} }}$, and thermal expansion $\frac{{\beta_{b} }}{{\beta_{f} }}$.

The heat capacity:

23 $$ Cr = \frac{{(\rho C_{p} )_{b} }}{{(\rho C_{p} )_{f} }} = \frac{\varphi }{\delta Ste}\left[ {\Lambda \frac{\pi }{2}\sin \left( {\frac{\pi }{\delta }(\theta - \theta_{f} + \frac{\delta }{2}} \right)} \right] + 1 - \varphi + \lambda \varphi , $$

with

24 $$ \Lambda = \left\{ {\begin{array}{*{20}l} 0 \hfill & { \theta < \theta_{f} - \frac{\delta }{2}} \hfill \\ 1 \hfill & {\left( {\theta_{f} - \frac{\delta }{2}} \right) < \theta < \left( {\theta_{f} + \frac{\delta }{2}} \right)} \hfill \\ 0 \hfill & {\theta > \theta_{f} + \frac{\delta }{2}} \hfill \\ \end{array} } \right., $$

where $$\theta_{f} = \frac{{T_{f} - T_{c} }}{\Delta T}, \delta = \frac{{T_{Mr} }}{\Delta T}, \lambda = \frac{{\left( {(C_{p} )_{c,l} + \chi (C_{p} )_{s} } \right)\rho_{s} \rho_{c} }}{{\left( {\rho_{s} + \chi \rho_{c} } \right)(\rho C_{p} )_{f} }}, Ste = \frac{{(\rho C_{p} )_{f} \Delta T(\rho_{s} + \chi \rho_{c} )}}{{\left( {1 + \chi } \right)h_{sf} \rho_{s} \rho_{c} }}$$.

The mean Nusselt number:

25 $$ \overline{Nu} = \frac{ - 1}{{L_{ctot} }}\mathop \int \limits_{0}^{{L_{ctot} }} \frac{{k_{m,b} }}{{k_{f} }}\frac{\partial \theta }{{\partial {\varvec{n}}}} d\xi , $$

where $L_{ctot}$ is a total length of the cold walls. ${\varvec{n}}$ is a normal vector. The references^5,53,57,58^ are summarizing the NEPCM and mixture fluid properties.

## ISPH method

The solver steps based on the time-fractional derivative are:

Step 1:

26 $$ U^{*} = U^{n} + \varepsilon \left( {\Delta \tau } \right)^{\alpha } \mathop \sum \limits_{k = 0}^{m} \left( { - 1} \right)^{k} \left( {\begin{array}{*{20}c} {1 - \alpha } \\ k \\ \end{array} } \right)\left( {\frac{{\mu_{b} }}{{\varepsilon \mu_{f} }}\frac{{\rho_{f} }}{{\rho_{b} }}\Pr \left( {\frac{{\partial^{2} U}}{{\partial X^{2} }} + \frac{{\partial^{2} U}}{{\partial Y^{2} }}} \right)^{n - k} - \frac{{\sigma_{b} }}{{\sigma_{f} }}\frac{{\rho_{f} }}{{\rho_{b} }} Ha^{2} \Pr \left( {U^{n - k} \sin^{2} \gamma - V^{n - k} \sin \gamma \cos \gamma } \right) - \frac{{\mu_{b} }}{{\mu_{f} }}\frac{{\rho_{f} }}{{\rho_{b} }}\Pr \frac{{U^{n - k} }}{Da} - \left( {\frac{C}{{\sqrt {Da} }}\frac{{\sqrt {U^{2} + V^{2} } }}{{\varepsilon^{\frac{3}{2}} }}} \right)^{n - k} U^{n - k} } \right), $$

27 $$ \begin{aligned}   V^{*}  &  = V^{n}  + \varepsilon \left( {\Delta \tau } \right)^{\alpha } \sum\nolimits_{{k = 0}}^{m} {( - 1)^{k} \left( {\begin{array}{*{20}c}    {1 - \alpha }  \\    k  \\   \end{array} } \right)} \left( {\frac{{\mu _{b} }}{{\varepsilon \mu _{f} }}\frac{{\rho _{f} }}{{\rho _{b} }}\Pr \left( {\frac{{\partial ^{2} V}}{{\partial X^{2} }} + \frac{{\partial ^{2} V}}{{\partial Y^{2} }}} \right)^{{n - k}} } \right. + \frac{{\left( {\rho \beta } \right)_{{nf}} }}{{\rho _{{nf}} \beta _{f} }}Ra\Pr \left( {\theta ^{{n - k}}  + N\Phi ^{{n - k}} } \right) \\     & \quad \left. { - \frac{{\sigma _{b} }}{{\sigma _{f} }}\frac{{\rho _{f} }}{{\rho _{b} }}Ha^{2} \Pr \left( {V^{{n - k}} \cos ^{2} \gamma  - U^{{n - k}} \sin \gamma \cos \gamma } \right) - \frac{{\mu _{b} }}{{\mu _{f} }}\frac{{\rho _{f} }}{{\rho _{b} }}\Pr \frac{{V^{{n - k}} }}{{Da}} - \left( {\frac{C}{{\sqrt {Da} }}\frac{{\sqrt {U^{2}  + V^{2} } }}{{\varepsilon ^{{\frac{3}{2}}} }}} \right)^{{n - k}} V^{{n - k}} ,} \right) \\  \end{aligned}  $$

Pressure Poisson equation (PPE):

28 $$ \left( {\Delta \tau } \right)^{\alpha } \mathop \sum \limits_{k = 0}^{m} \left( { - 1} \right)^{k} \left( {\begin{array}{*{20}c} {1 - \alpha } \\ k \\ \end{array} } \right) \nabla^{2} P^{n - k + 1} = \frac{1}{\varepsilon }\frac{{\rho_{b} }}{{\rho_{f} }}\left( {\frac{{\partial U^{*} }}{\partial X} + \frac{{\partial V^{*} }}{\partial Y}} \right) + \gamma \frac{{\left( {\rho_{f} - \rho^{num} } \right)}}{{\rho_{f} \left( {\Delta \tau } \right)^{\alpha } }}, $$

Corrected velocities:

29 $$ U^{n + 1} = U^{*} - \left( {\Delta \tau } \right)^{\alpha } \mathop \sum \limits_{k = 0}^{m} \left( { - 1} \right)^{k} \left( {\begin{array}{*{20}c} {1 - \alpha } \\ k \\ \end{array} } \right)\frac{{\varepsilon \rho_{f} }}{{\rho_{b} }}\left( {\frac{\partial P}{{\partial X}}} \right)^{n - k + 1} , $$

30 $$ V^{n + 1} = V^{*} - \left( {\Delta \tau } \right)^{\alpha } \mathop \sum \limits_{k = 0}^{m} \left( { - 1} \right)^{k} \left( {\begin{array}{*{20}c} {1 - \alpha } \\ k \\ \end{array} } \right)\frac{{\varepsilon \rho_{f} }}{{\rho_{b} }}\left( {\frac{\partial P}{{\partial X}}} \right)^{n - k + 1} , $$

The thermal equation is:

31 $$ \theta^{n + 1} = \theta^{n} + \left( {\Delta \tau } \right)^{\alpha } \mathop \sum \limits_{k = 0}^{m} \left( { - 1} \right)^{k} \left( {\begin{array}{*{20}c} {1 - \alpha } \\ k \\ \end{array} } \right)\frac{{k_{m,b} }}{{k_{f} \left( {\varepsilon CR + \left( {1 - \varepsilon } \right)\frac{{\left( {\rho C} \right)_{s} }}{{\left( {\rho C} \right)_{f} }}} \right)}}\left( {\frac{{\partial^{2} \theta }}{{\partial X^{2} }} + \frac{{\partial^{2} \theta }}{{\partial Y^{2} }}} \right)^{n - k} , $$

Updated positions are:

32 $$ X^{n + 1} = X^{n} + \left( {\Delta \tau } \right)^{\alpha } \mathop \sum \limits_{k = 0}^{m} \left( { - 1} \right)^{k} \left( {\begin{array}{*{20}c} {1 - \alpha } \\ k \\ \end{array} } \right) U^{n - k + 1} , $$

33 $$ Y^{n + 1} = Y^{n} + \left( {\Delta \tau } \right)^{\alpha } \mathop \sum \limits_{k = 0}^{m} \left( { - 1} \right)^{k} \left( {\begin{array}{*{20}c} {1 - \alpha } \\ k \\ \end{array} } \right) V^{n - k + 1} , $$

The shifting technique^59^ is:

34 $$ {\mathcal{F}}_{{i^{\prime } }} = {\mathcal{F}}_{i} + \left( {\nabla {\mathcal{F}}} \right)_{i} \cdot \delta {\varvec{r}}_{{ii^{\prime } }} + {\mathcal{O}}\left( {\delta {\varvec{r}}_{{ii^{\prime } }}^{2} } \right), $$

35 $$ \delta {\varvec{r}}_{{ii^{\prime } }} = - {\mathcal{D}} \nabla C_{i}^{\prime } . $$

The solver steps of the ISPH method are implemented by an in-house FORTRAN 90 code. The calculations are performed employing SHAHEEN-II owned by King Abdullah University of Science and Technology (KAUST), Jeddah, Saudi Arabia. Calculation of one cycle of the dual rotations between an inner fin and outer domain is taken around ($\tau \approx 1.2)$, which is elapsed 72 h in the PC-Cluster of SHAHEEN-II.

## Verification tests

**Figure 2:**
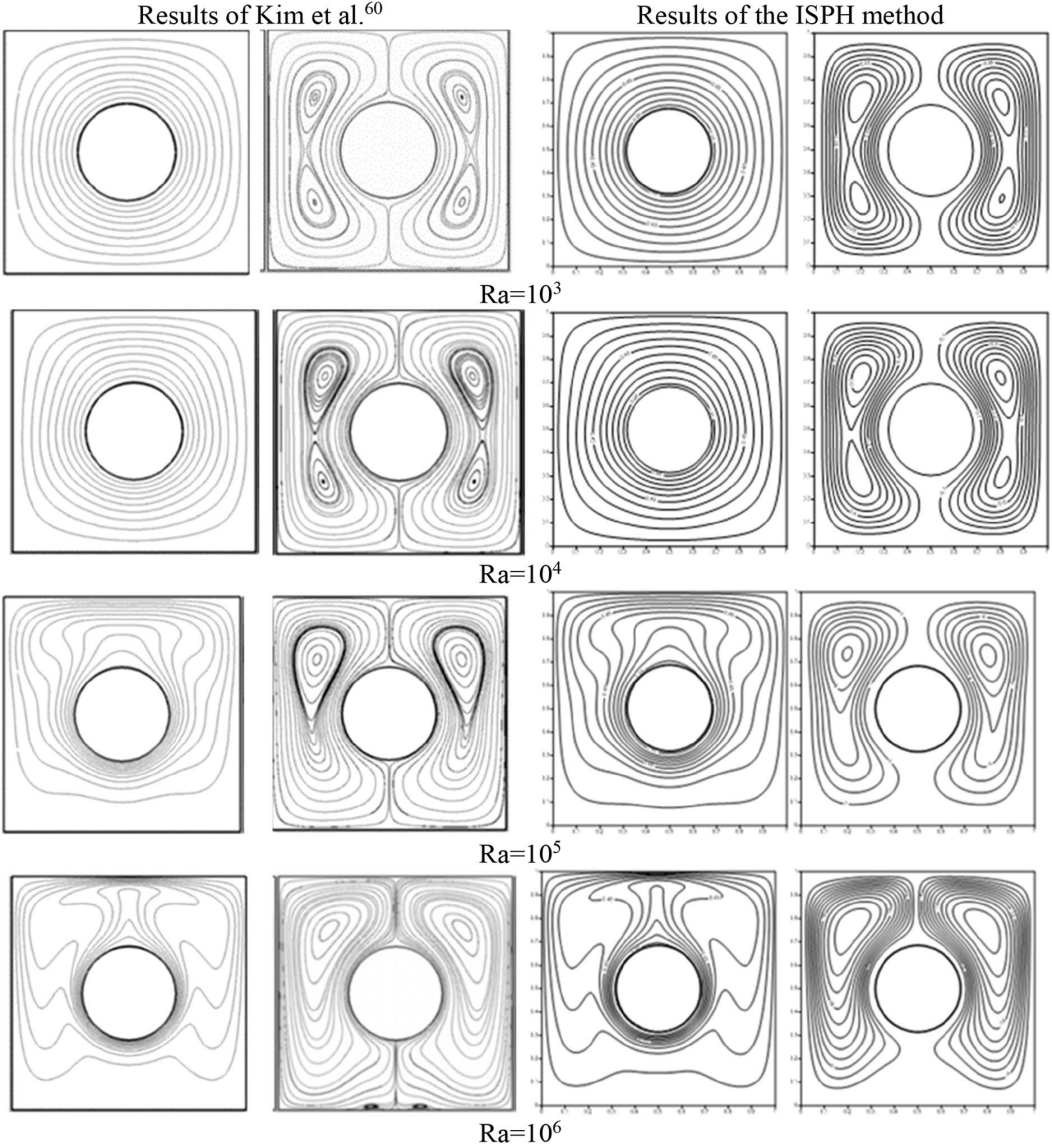
Comparison of the isotherms and streamlines between the results of Kim et al.^60^ and the ISPH method at Rayleigh number $Ra = 10^{3} , 10^{4} , 10^{5}$ and $10^{6}$.

This section checks the efficiency of the ISPH method in simulating the natural convection resultant from an inner circular cylinder. Figure 2 shows the comparison of the isotherms and streamlines between the results of Kim et al.^60^ and the ISPH method at Rayleigh number $Ra = 10^{3} , 10^{4} , 10^{5}$ and $10^{6}$. It is remarked that the present results of the ISPH method agree well the reference of Kim et al.^60^. The results are presented at the steady-state and convergence criteria for the current verification is taken as $10^{ - 6}$. Further studies on the verification tests of the natural/mixed convection flow using the ISPH method can be found in references^61–65^.

**Figure 3:**
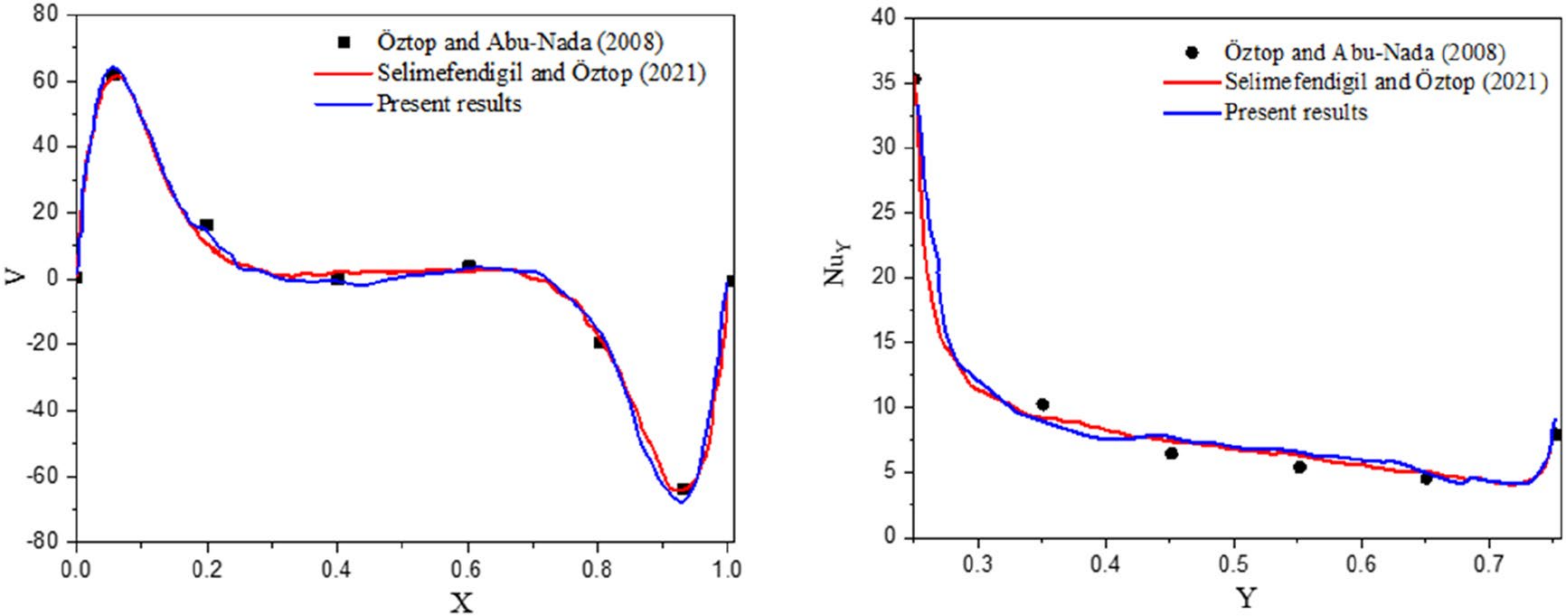
The verification code of the ISPH method with the results of Oztop and Abu-Nada^66^ and Selimefendigil and Öztop^15^.

Further validation of the ISPH method with the numerical results of for natural convection in a cavity at Rayleigh number $Ra = 10^{5}$ is shown in Fig. 3. The validation results are providing adequate confidence of the ISPH method.

## Results and discussion

The current research aims to apply a fractional time derivative in the ISPH method for emulating the dual rotation between an inner cross fin and outer hexagonal-shaped domain. The hexagonal-shaped cavity is containing two rectangle heat sources on the flat walls and is suspended by NEPCM. During the executed simulations, the parameters are fixed at dimensionless angular velocity $\omega = 2.5$, a porosity parameter $\varepsilon = 0.6$, Stefan number $Ste = 0.2$, and a magnetic inclination angle $\gamma = 45^\circ$. The influences of a fractional time derivative $\left( {0.92 \le \alpha \le 1} \right)$, Hartmann number $\left( {0 \le Ha \le 80} \right)$, the fin length $\left( {0.2 \le L_{Fin} \le 1} \right)$, Darcy parameter $\left( {10^{ - 2} \le Da \le 10^{ - 4} } \right)$, Rayleigh number $\left( {10^{3} \le Ra \le 10^{6} } \right)$, a fusion temperature $\left( {0.05 \le \theta_{f} \le 0.8} \right)$, and solid volume fraction $\left( {0 \le \varphi \le 0.06} \right)$ on the velocity field, isotherms, and mean Nusselt number $\overline{Nu}$ are discussed. The dimensionless angular velocity is lowering at $\omega = 2.5$ to consider the natural convection mode only during the simulations.

**Figure 4:**
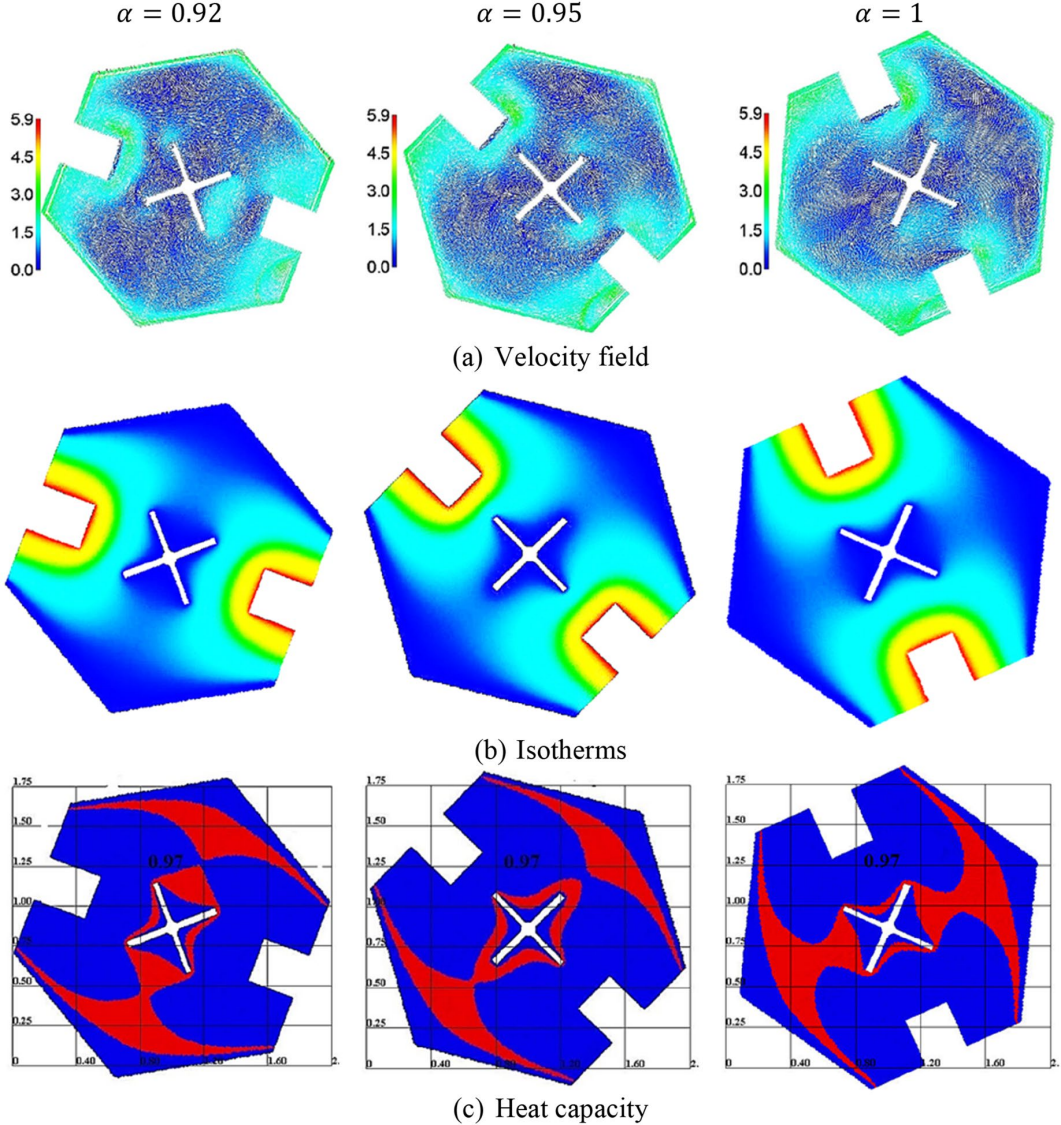
The velocity field, isotherms, and a heat capacity under the variations of a fractional time derivative $\alpha$ at $Ra = 10^{4} , \;Da = 10^{ - 3} ,\; \varphi = 0.05, \;Ha = 20,\; \varepsilon = 0.6, \;Ste = 0.2, \;\tau = 0.16, \;\theta_{f} = 0.05\; {\text{and}}\;\gamma = 45^\circ$.

Figure 4 shows the velocity field, isotherms, and heat capacity under the variations of a fractional time derivative $\alpha .$ It is remarked that the dual rotation between an inner fin and outer hexagonal-shaped domain is affected by the variations on a fractional time derivative $\alpha$. Consequently, the velocity field, and isotherms are varied according to the variations on $\alpha$. Thus, the zone of a phase change material (PCM) is influenced by the location of the outer domain and inner shape. The current investigation reported that the PCM is changed as the fractional time derivative $\alpha$ is varied. Here, the factor $\alpha$ is playing a significant role in controlling the rotation speed between inner/outer shapes, nanofluid movements, and heat transfer in a hexagonal-shaped cavity.

**Figure 5:**
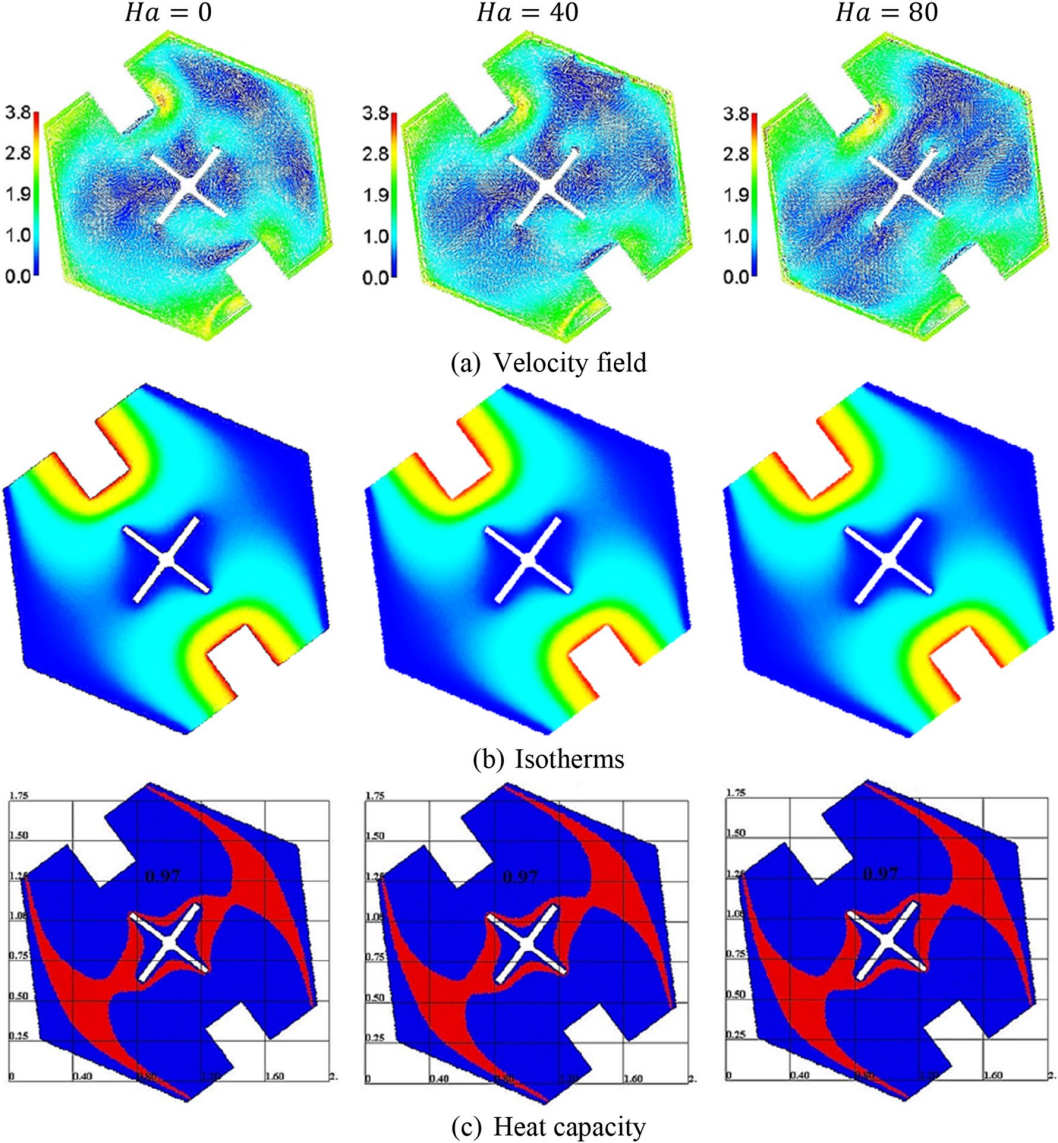
The velocity field, isotherms, and a heat capacity under the variations of the Hartmann number $Ha$ at $Ra = 10^{4} , \;Da = 10^{ - 3} , \;\alpha = 0.97, \;\varphi = 0.05, \;\varepsilon = 0.6, \;Ste = 0.2, \;\tau = 0.16, \;\theta_{f} = 0.05 \;{\text{and}}\;\gamma = 45^\circ$.

Figure 5 signifies the impacts of Hartmann number $Ha$ on the velocity field, isotherms, and heat capacity. Physically, an increase in Hartmann number reduces the convection flow and suppresses the fluid flow due to the Lorentz force. In this model, due to the presence of a cold fin in the cavity’s center and the two rectangle heaters in the flat walls, the contributions of the Hartmann number are less. As a result, an increment in $Ha$ is giving a minor impact on the velocity field, isotherms, and heat capacity within a cavity.

**Figure 6:**
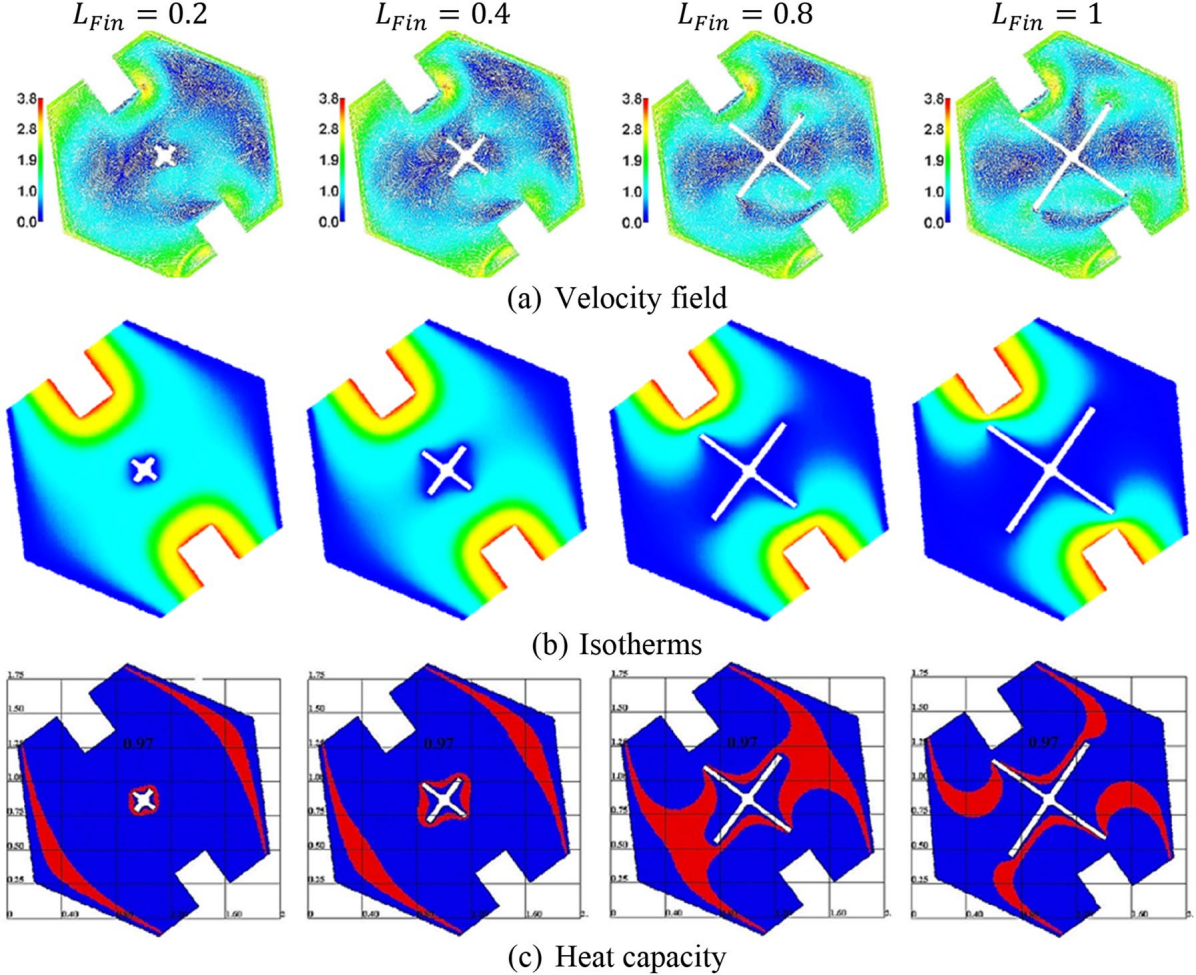
The velocity field, isotherms, and a heat capacity under the variations of the fin length $L_{Fin}$ at $Ra = 10^{4} , \;Da = 10^{ - 3} , \;\alpha = 0.97, \;\varphi = 0.05,\;Ha=20, \;\varepsilon = 0.6, \;Ste = 0.2, \;\tau = 0.16, \;\theta_{f} = 0.05 \;{\text{and}}\;\gamma = 45^\circ$.

Figure 6 indicates the effects of the fin length $L_{Fin}$ on the velocity field, isotherms, and heat capacity. The inner fin acts as a prominent character in the cooling process inside a hexagonal-shaped cavity, the variations on the fin length $L_{Fin}$ are changing the nanofluid movements in a cavity. As the cool fin represents a blockage within a hexagonal-shaped cavity, an increase in $L_{Fin}$ declines the velocity field. Increasing the fin length $L_{Fin}$ augments the cooling area and accordingly the temperature distributions are reduced. The PCM is affected clearly by the variations in the fin length $L_{Fin}$. It is observed that the expansion in the fin length controls the location of a phase change zone.

**Figure 7:**
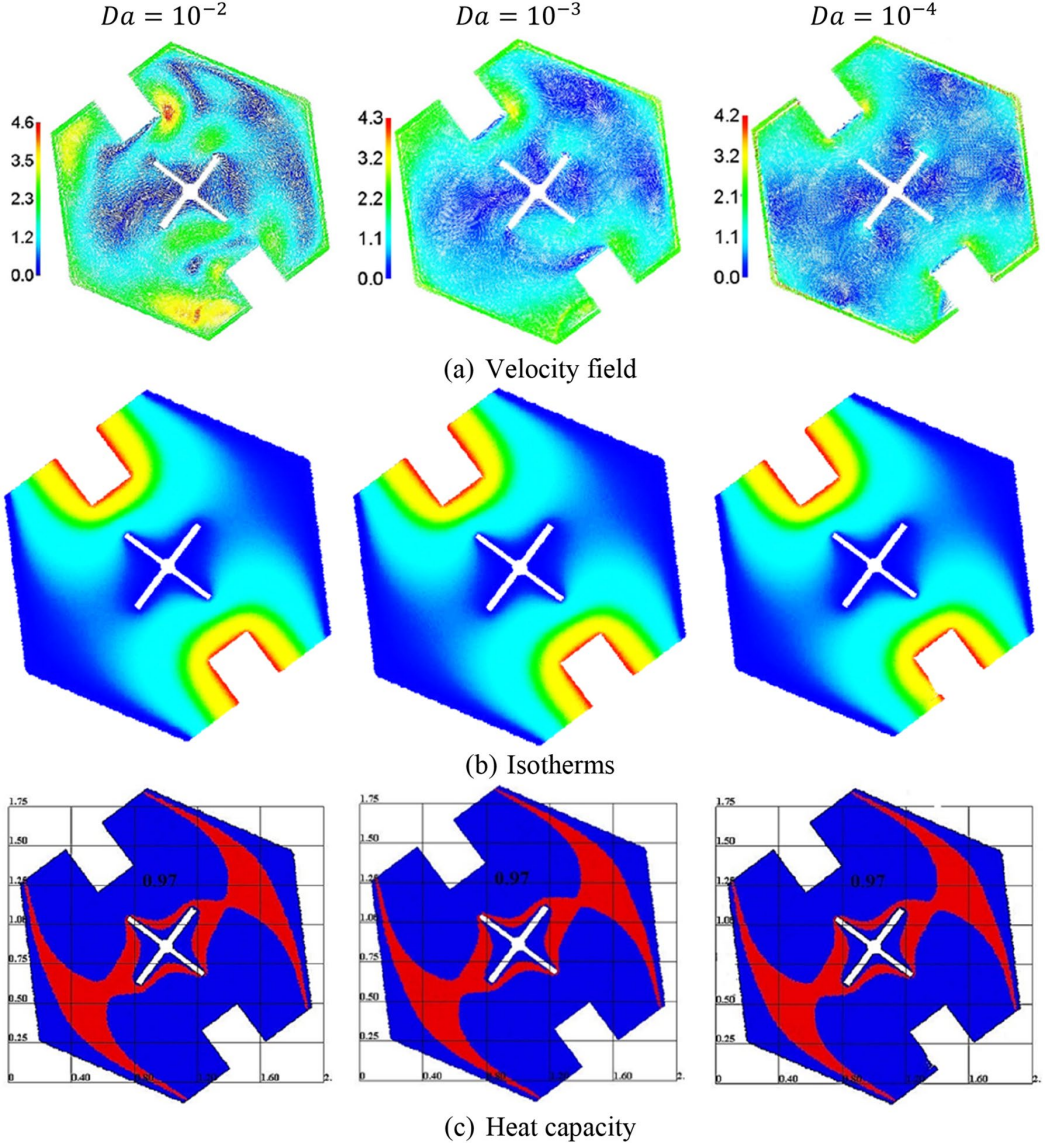
The velocity field, isotherms, and heat capacity under the variations of Darcy parameter $Da$ at $Ra = 10^{4} , \;\alpha = 0.97,\; \varphi = 0.05, \;Ha = 20, \;\varepsilon = 0.6, \;Ste = 0.2, \;\tau = 0.16, \;\theta_{f} = 0.05 \;{\text{and}}\;\gamma = 45^\circ$.

Figure 7 introduces the influences of Darcy parameter $Da$ on the velocity field, isotherms, and heat capacity. Physically, the Darcy parameter signifies the major element of the porous resistance for the fluid flow. Decreasing $Da$ from $10^{ - 2}$ to $10^{ - 4}$, leads to a decrease in the velocity’s maximum by 8.69%. Further, a decrease in $Da$ leads to a minor reduction in the temperature distributions. Thus, due to the minor change in the temperature distributions below the variations on $Da$, the phase change zone is affected slightly by variations on $Da$.

**Figure 8:**
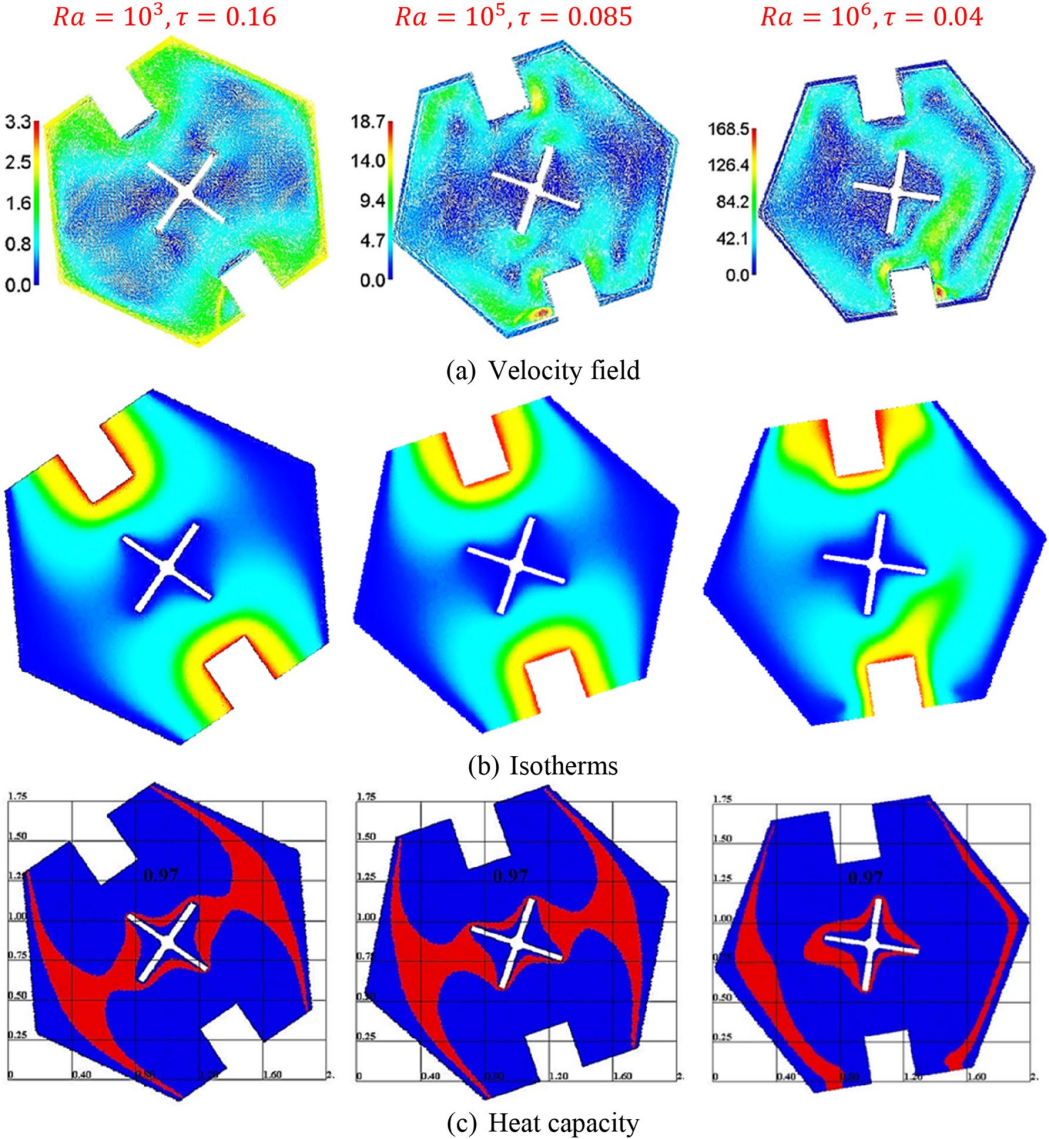
The velocity field, isotherms, and heat capacity under the variations of Rayleigh number $Ra$ at $Da = 10^{ - 3} , \;\alpha = 0.97, \;\varphi = 0.05, \;Ha = 20, \;\varepsilon = 0.6, \;Ste = 0.2, \;\theta_{f} = 0.05 \;{\text{and}}\;\gamma = 45^\circ$.

Figure 8 shows the velocity field, isotherms, and heat capacity under the variations of Rayleigh number $Ra$. Physically, the Rayleigh number augments the buoyancy forces which powers the fluid flow and heat transfer within a cavity. The strength of the velocity field is increasing strongly as $Ra$ increases. Further, a growth in $Ra$ strengthens the temperature distributions in a hexagonal-shaped cavity. Thus, the heat capacity is affected clearly by an increase in $Ra$. The physical meaning of a high $Ra$ is powering the buoyancy-driven flow.

**Figure 9:**
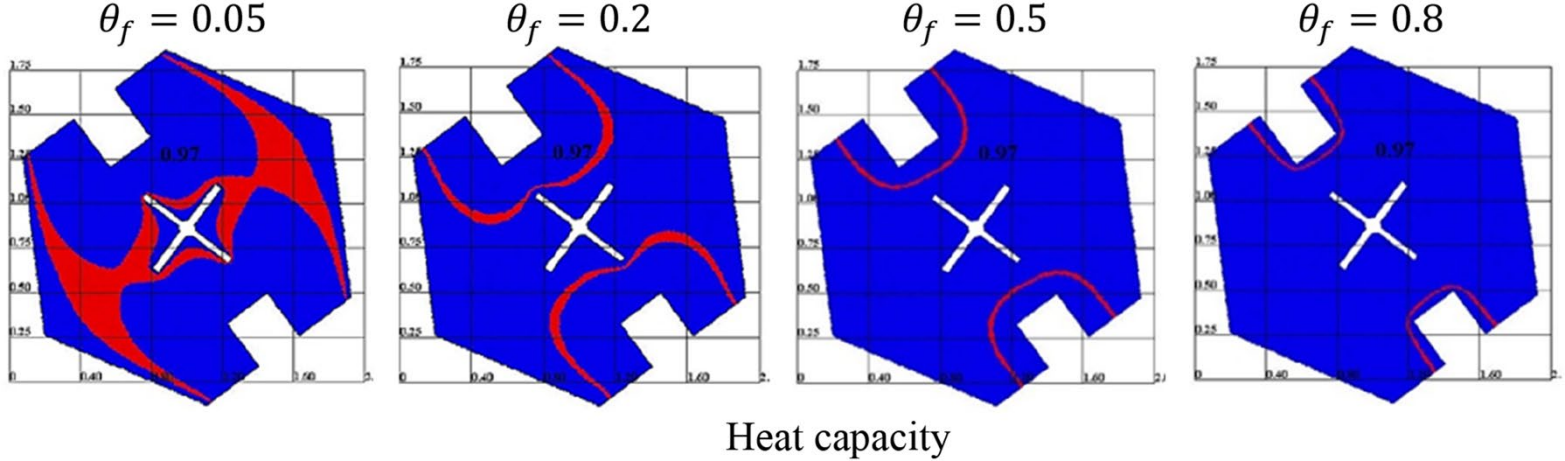
The heat capacity under the variations of a fusion temperature $\theta_{f}$ at $Ra = 10^{4} , \;Da = 10^{ - 3} , \;\alpha = 0.97, \;\varphi = 0.05, \;Ha = 20,\; \varepsilon = 0.6, \;Ste = 0.2, \;\tau = 0.16 \;{\text{and}} \;\gamma = 45^\circ$.

**Figure 10:**
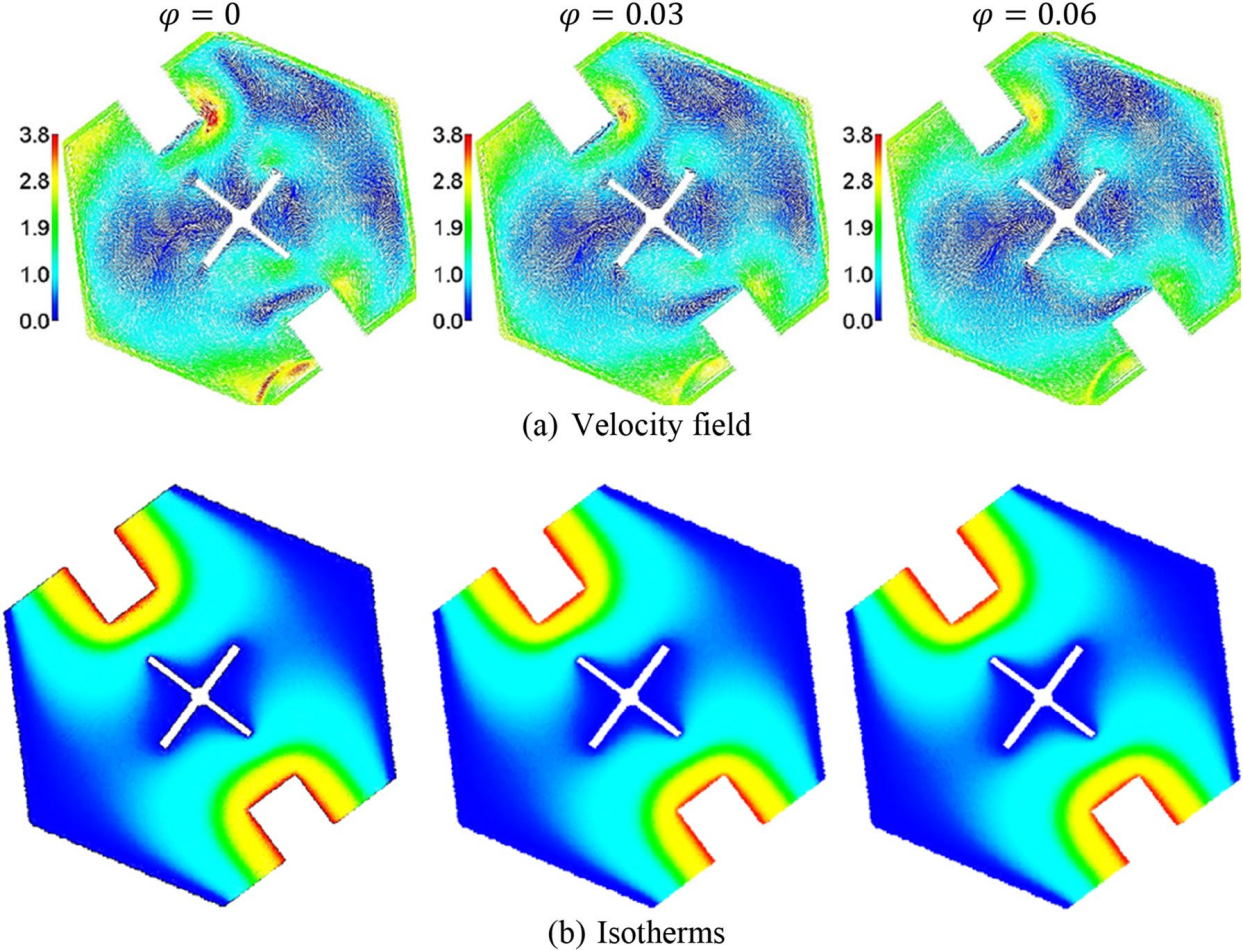
The velocity field, and isotherms under the variations of the solid volume fraction $\varphi$ at $Ra = 10^{4} , \;Da = 10^{ - 3} , \;\alpha = 0.97,\; Ha = 20, \;\varepsilon = 0.6, \;Ste = 0.2, \;\tau = 0.16, \;\theta_{f} = 0.05 \;{\text{and}}\;\gamma = 45^\circ$.

Figure 9 presents the fusion temperature $\theta_{f}$ impacts on the heat capacity. It is remarked that an increment of $\theta_{f}$ reduces a phase change zone. Further, increasing in $\theta_{f}$ closes the phase change zone near the rectangle heaters of a hexagonal-shaped cavity. The physical explanation returns to the connection between a heat capacity $Cr$ and a fusion temperature $\theta_{f}$. Figure 10 introduces the impacts of a solid volume fraction $\varphi$ on the contours of the velocity field, and isotherms. Physically, adding more concentrations of the nanoparticles boosts the viscosity of the mixture fluid, and accordingly, the velocity field is decreasing. There are minor changes in the isotherms according to adding more concentrations of the nanoparticles. The fewer contributions of $\varphi$ on the heat transfer return to the presence of a cool fin inside a hexagonal-shaped cavity.

**Figure 11:**
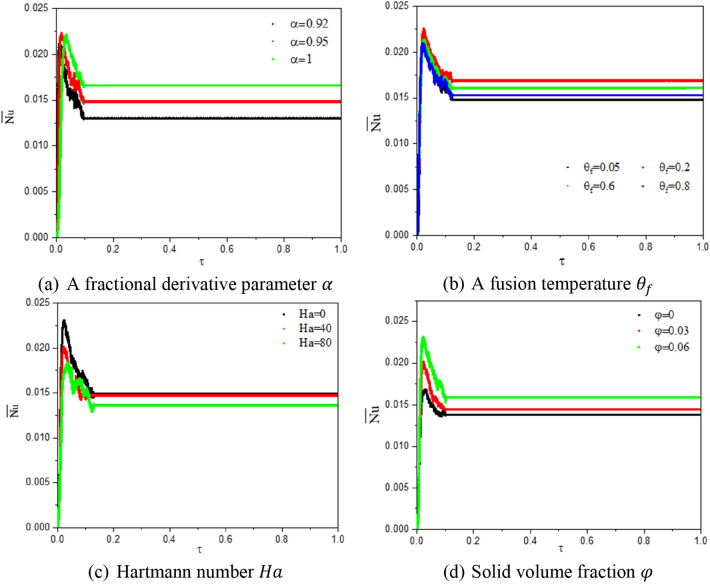
Profiles of $\overline{Nu}$ under the effects of a fractional derivative parameter $\alpha$, a fusion temperature $\theta_{f}$, Hartmann number $Ha$, and solid volume fraction $\varphi \;{\text{at}}\;Ra = 10^{4} , \;Da = 10^{ - 3} ,\; \varepsilon = 0.6, \;Ste = 0.2, \;{\text{and}}\;\gamma = 45^\circ$.

Figure 11 indicates the profiles of $\overline{Nu}$ under the effects of a fractional derivative parameter $\alpha$, a fusion temperature $\theta_{f}$, Hartmann number $Ha$, and solid volume fraction $\varphi$. Initially, the profiles of $\overline{Nu}$ are fluctuating under the effects of pertinent parameters due to the dual rotation between an inner fin and outer hexagonal-shaped domain at the transition state. It is seen that the profiles of $\overline{Nu}$ are affected by the variations on the pertinent parameters. It is seen that a fractional derivative $\alpha = 1$ gives the highest values of $\overline{Nu}$. Second, the value of $\theta_{f} = 0.2$ provides the highest values of $\overline{Nu}$ and the tendency of $\overline{Nu}$ is fluctuating below the variation of $\theta_{f}$. Third, the values of $\overline{Nu}$ are decreasing according to an expansion in the Hartmann number. Fourth, adding more concentration of nanoparticles is enhancing the values of $\overline{Nu}$.

## Conclusion

The originality of the study is emulating the natural convection of NEPCM embedded in a hexagonal-shaped cavity under the impacts of a magnetic field and dual rotation between an inner fin and outer hexagonal-shaped domain. The ISPH method is developed by including the time-fractional derivative based on the Grunwald–Letnikove derivative in the solving steps for conducting the current physical problem. The executed simulations indicated that the variations on the fractional time derivative are changing the dual rotation amongst the inner fin and outer domain. As a result, the nanofluid movements, heat transfer, and phase change material within a hexagonal-shaped cavity are affected by the variations of a fractional time derivative. The inserted cool fin is functioning efficiently in the cooling process and adjusting the phase change zone within a hexagonal-shaped cavity. Increasing the fin length augments the cooling area and controls the location of a phase change zone. The velocity’s maximum reduces by 8.69% as Darcy parameter declines from $10^{ - 2}$ to $10^{ - 4}$. A growth in Rayleigh number strengthens the nanofluid movements and temperature allotments inside a hexagonal-shaped cavity. A fusion temperature adjusts the power and place of a phase change zone. The highest values of $\overline{Nu}$ are obtained at $\alpha = 1$. An expansion in the Hartmann number reduces the values of $\overline{Nu}$. Adding more concentration of nanoparticles is improving the values of $\overline{Nu}$.

## Future work

In future work, the fractional-time derivative will adopt the most recent formulation in the fractional calculus. More respective studies in the fractional-space derivative will be researched.

## List of symbols

$$B_{o}$$: Magnetic field
$$C_{p}$$: Specific heat, $$\left( {{\text{J}}\;{\text{kg}}^{ - 1} \;{\text{K}}^{ - 1} } \right)$$
$$Cr$$: Heat capacity
$$D_{t}^{\alpha }$$: Fractional derivative
$$Da$$: Darcy parameter, $$\left( {\frac{K}{{L_{ }^{2} }}} \right)$$
$${\text{g}}$$: Gravitational acceleration, $$\left( {{\text{m/s}}^{2} } \right)$$
$$Ha$$: Hartmann number, $$ \left( {B_{0} L\sqrt {\frac{{\sigma_{f } }}{{\mu_{f} }}} } \right)$$
$$k$$: Thermal conductivity, $$\left( {{\text{W}}\;{\text{m}}^{ - 1} \;{\text{K}}^{ - 1} } \right)$$
$$K$$: Permeability
$L$: Length of a cavity, (m)
$L_{fin}$: Length of fin, (m)
$L_{h}$: Length of the heated rectangle, (m)
$$Nu$$: Nusselt number
$\overline{Nu}$: Mean Nusselt number
$$Pr$$: Prandtl number, $$\left( {\nu_{f} {/}\zeta_{f} } \right)$$
$$p$$: Pressure, $$\left( {{\text{Nm}}^{ - 2} } \right)$$
$$Ra$$: Rayleigh number, $$\left( {\frac{{g\beta_{f} \left( {T_{h} - T_{c} } \right)L^{3} }}{{\nu_{f} \zeta_{f} }}} \right)$$
$$T$$: Temperature, $$\left( {\text{K}} \right)$$
$$t$$: Time, (s)
$$u$$: Velocity vector, (m/s)
$$u, v$$: Velocity components, (m/s)
$$U, V$$: Dimensionless velocity
$$x, y$$: Cartesian coordinates, m
$X, Y$: Dimensionless Cartesian coordinates
$\chi$: Core–shell weight ratio

## Greek symbols

$$\zeta$$: Thermal diffusivity, $$\left( {{\text{m}}^{2} \;{\text{s}}^{ - 1} } \right)$$
$\alpha$: Afractional time derivative
$\beta$: Coefficient of thermal expansion $\left( {{\text{K}}^{ - 1} } \right)$
$$\delta$$: Heat parameter
$\phi$: Nanoparticle volume fraction
$${\Gamma }$$: Gamma function
$$\varepsilon$$: Porosity
$$\mu$$: Viscosity
$\gamma$: A magnetic field inclination angle
$$\nu$$: Kinematic viscosity, $$\left( {{\text{m}}^{2} \;{\text{s}}^{ - 1} } \right)$$
$$\rho$$: Density, $$\left( {{\text{kg/m}}^{3} } \right)$$
$$\sigma$$: Capacity ratio
$$\tau$$: Dimensionless time
$$\theta$$: Dimensionless temperature
$$\theta_{f}$$: Fusion temperature
${\upomega }$: Dimensionless angular velocity

## Subscripts

$$f$$: Fluid
$b$: Bulk properties of the suspension
hex: Hexagonal
$$s$$: Solid
$$fin$$: Inner fin
